# On the Use of Aconite as a Therapeutic Agent

**Published:** 1857-11

**Authors:** Edward B. Stevens

**Affiliations:** Cincinnati


*On the use of Aconite as a Therapeutic Agent. By Edward B. Stevens, M.D., Cincinnati.*

I have found good results from the use of aconite, in almost the entire range of neuralgic affections, and in those obscure complications of rheumatism and neuralgia in which there is freedom from local or constitutional trouble, independent of the nervous derangement; but more particularly in such cases as are usually styled pure neuralgia, I have frequently had results almost as prompt and satisfactory as were recently attributed to valerianate of ammonia.

Something more than a year ago, a lady called upon me for advice, for a severe neuralgia of the face and head. From the history of the case, I supposed it to be a result of previous attacks of miasmatic disease, and accordingly prescribed quinine, which relieved her temporarily, but every few weeks she would have a relapse, and for several days at a time suffer excruciatingly. In addition to the quinine, quite a variety of customary treatment was resorted to, until in November I directed the following prescription, after she had suffered a week, and had tried without avail all the remedies that hitherto had given temporary relief:

℞. Tinct. Aconite *Root*, ʒi.,

Tinct. Cimicifuga, ℥ij.

Sig.—To take a teaspoonful every four hours.

My patient took three doses, when she was promptly and entirely relieved; and, what is better, she has scarcely felt a neuralgic twinge since, now about ten months.

In another case, a friend had for a long time suffered a peculiar form of neuralgia, or neuralgic rheumatism in the arm, which seemed to yield to no remedy, even temporarily. I suggested the aconite. The result was equally prompt with that of the case I have just given. I might multiply these satisfactory examples to considerable extent.

The formula given above is the one I most frequently use in administering the aconite to adults. It will be observed that, given in that way, each dose would be equivalent to about 4 drops of the tincture (except that tinct. aconite gives something more than 60 drops to the drachm), and in that dose I have never seen any effects sufficiently marked or violent to occasion alarm. It will also be observed that the tinct. of the *root* is directed. The U. S. Dispensatory recognises two officinal tinctures: of the *leaves* and of the *root*. I prefer the tinct. of the root, as of greater efficiency, and more reliable as to uniformity of effect. The tinct. cimicifuga is intended chiefly as a vehicle, but selected with the view to its contributing to the special effect of the aconite.

I have not tried the aconite in acute rheumatism, but in the chronic rheumatic pains, particularly such as aged people complain of, I have seen very excellent effects. Neither have I used it much locally, though with many, even those who do not use it internally, it is a favorite topical application.

There is a form of neuralgia, associated with uterine derangement, which I have frequently met with, coming up sometimes in connection with the catamenial period through the hips, sacrum and uterine region. Sometimes I have seen this group of symptoms succeed abortion. I remember a case of this kind where the local distress I have just alluded to remained very troublesome for several weeks, while much of the time there was almost uninterrupted sleeplessness, despite the free use of opiates. The tinct. aconite root, given through the afternoon and evening, relieved the neuralgic pain, and secured a sweet and refreshing sleep through the night.—*Cin. Med. Observer.*

